# Influence of Inertial Vlasov Foundation Parameters on the Dynamic Response of the Bernoulli—Euler Beam Subjected to A Group of Moving Forces—Analytical Approach

**DOI:** 10.3390/ma15093249

**Published:** 2022-04-30

**Authors:** Magdalena Ataman, Wacław Szcześniak

**Affiliations:** Faculty of Civil Engineering, Warsaw University of Technology, 16 Armii Ludowej Ave., 00-637 Warsaw, Poland; w.szczesniak@il.pw.edu.pl

**Keywords:** Bernoulli–Euler beam, inertial Vlasov soil model, moving force, moving group of forces, forced and free vibrations, analytical solution

## Abstract

The subject of this study is the vibration of the Bernoulli–Euler beam on a three-parameter inertial foundation caused by a group of moving forces. The solution to the problem is obtained analytically. The influence of deformable foundation properties on the dynamic response of the beam in the case of forced vibration and the case of free vibration after the load has left the beam is analysed. The influence of velocity on the dynamic response of the beam is also investigated in both cases. The results can be used as a benchmark for calculating more complex engineering structures under moving loads caused by road or railroad vehicles. The results of the investigation are presented in the figures. It is evident that the coefficient determining the foundation inertia has a significant influence on the dynamic deflection of the beam. Taking shear into account in the Vlasov foundation model has little effect on the dynamic deflections of the beam. The equivalent damping number introduced into the Kelvin–Voigt model takes into account the structure damping and mass damping of the beam.

## 1. Introduction

Vibrations of beams and plates under moving loads are closely related to the dynamics of engineering structures: bridges, roads, airport pavements, and railway pavements. The problem of moving loads on beams dates back to the mid-19th century; it is one of the oldest problems in structural dynamics. Numerous monographs, review papers, and original scientific studies on the subject can be found in the literature. A literature review on moving loads is presented, inter alia, in [1]. It includes studies which present analytical solutions and works using computer methods to solve the problem. The comprehensive book on this subject is the monograph by Fryba [2].

**Figure 1:**
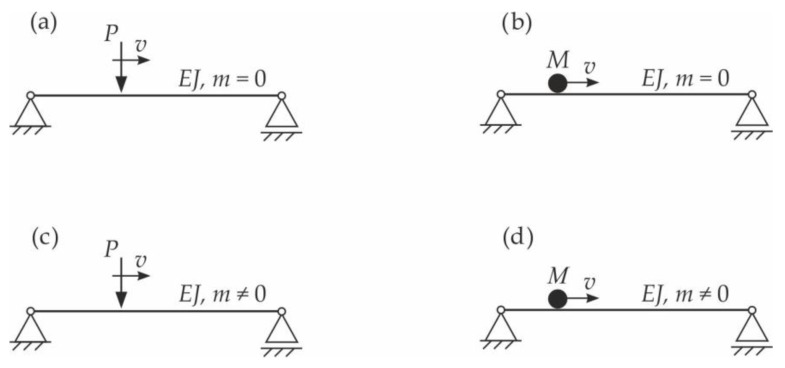
Dynamic diagrams of a simply supported beam under non-inertial and inertial moving loads: (**a**) the problem of structural mechanics (influence line), (**b**) the problem of Willis–Stokes (1849), (**c**) the problem of Kriloff (1905), and (**d**) the problem of Inglis (1934) and Renaudot (1861) [3].

The literature on the dynamics of beams under moving loads is extremely extensive. Traditionally, authors have investigated four types of problems related to inertial and non-inertial moving loads [3] (Figure 1). The first studies assumed that the entire mass of the beam was concentrated in the beam mid-span or was completely neglected (Stokes’ problem). In 1905, Kriloff [4] solved the problem of a simply supported beam with a distributed mass under force moving at a constant speed. In 1934, an extensive work by Inglis [5] was published, in which a particle moving along a simply supported beam was considered. After this period, various studies observed that the moving load model is complex. It can be, for example, a one- or a two-mass oscillator with or without damping, e.g., [6,7].

Various beam theories are considered in the works related to moving loads: Bernoulli–Euler, Rayleigh, Flügge, Donell, or Timoshenko theories. For example, numerous papers deal with the Timoshenko beam under moving loads, including [8,9,10]. Some of the works refer to the Timoshenko beam resting on various deformable foundations, e.g., [11,12,13,14,15,16,17]. However, in most studies available in the literature, the foundation inertia is neglected. In the paper [18], the analysis of the critical velocity of a beam on a viscoelastic foundation under a moving load is provided for Euler–Bernoulli and Timoshenko–Rayleigh beam theories.

These numerous publications dealt with an analytical approach and problem solving using the modal analysis method, the eigenfunctions method, and the integral transformations method. Authors also used approximate methods when solving this type of problem, e.g., the Rayleigh method, the Bubnov–Galerkin method, and others.

Another approach is to use a discrete system model and finite element analysis. Modern computer methods lead to extensive vehicle models with several dozen degrees of freedom. At the same time, the supporting girders (beams, trusses, plates, and arches) often have several thousand degrees of freedom. 

Basic models can be used for the initial verification of calculations of complex structures by the finite element method using large computer systems. In [3], it was shown that the critical forces causing the buckling of the beam on the foundation, as well as the critical speed, not only depend on the weight of the beam but also on the mass of the moving load, the mass of the co-vibrating ground, and the shear coefficient of the soil. The model which takes into account these factors is the three-parameter (modified) Vlasov model [19]. In addition, the adoption of the Vlasov foundation three-parameter model of the inertial, deformable foundation allows examination of the impact of subgrade settlements deep into the foundation layer and in the beam vicinity.

A literature review on beams and plates on elastic foundations is presented in [20,21].

In the literature, there are many studies on the Vlasov non-inertial soil model and on the beams and plates resting on this foundation e.g., [22,23,24,25,26,27,28].

The beginning of analyses related to the search for a soil model taking into account the inertia of the subgrade dates back to the mid-20th century. Saito and Murakami [29] introduced separate insulated mass columns in the Winkler layer on a rigid base. Inertial models of the subgrade were also developed by Vlasov and Leontev [19], as well as Martinček [30]. The models taking into account the soil inertia were analysed in many publications, e.g., Rades [31], and Holder and Michalopoulos [32]. Few studies in the related literature deal with the analytical solution of vibration of beams on a three-parameter inertial Vlasov foundation.

Currently, computer methods (FEM and others [33,34,35]) allow consideration of the ground inertia, as well as the mass, speed, and acceleration of the vehicle in complex engineering structures. However, these are approximate methods and require verification and validation. Analytical solutions are used for this purpose. These are solutions that describe the engineering problems in a simplified manner. They also have some limitations. For example, they cannot be used for complex boundary conditions. However, their advantage over numerical methods is that they are strict methods. They also allow for a quick, complete analysis of structure vibrations as a function of soil parameters, load, and speed.

In this paper, the problem of a beam on Vlasov’s inertial foundation loaded with a group of moving forces is discussed and solved analytically. The impact of deformable foundation properties on the dynamic response of the beam is analysed in the case of forced vibration and in the case of free vibration (after the load has left the beam). The results can be used as a benchmark for calculating structures under moving loads caused by road or railroad vehicles.

The presented method also has practical application in the design of engineering structures exposed to vibrations caused by moving loads. It is useful in solving wave problems and essential in designing railroads adapted to high-speed trains. The work can also be used to design soil-steel shell structures carrying road and railroad traffic [36,37,38,39]. These structures have recently become widely used due to the ease of implementation and for economic reasons. The work can also be used in the analysis of seismic problems.

The study indicates that soil inertia cannot be neglected in dynamics.

## 2. Vlasov Soil Model

The Vlasov model of soil response is a two- or three-parameter elastic model, which is derived by introducing displacement constraints that simplify the basic equations for linear elastic isotropic continuum. A soil response function is obtained by imposing certain restrictions upon the possible distribution of displacements in an elastic layer.

**Figure 2:**
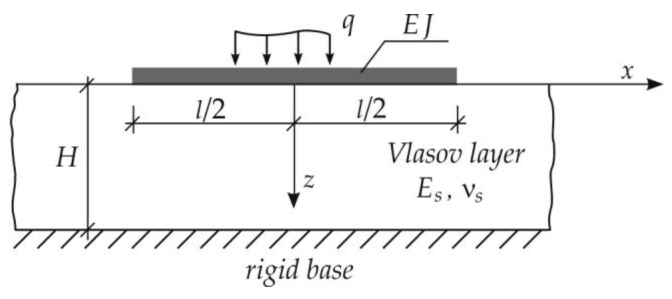
Beam on the Vlasov soil model.

When the linear problem (Figure 2) is considered, assumptions concerning the displacement components in the foundation layer are:

(1) under the beam, for −l/2<x<l/2
  (1) $$u_{x}=0,\;u_{y}=0,\;w\left(x,z,t\right)=w\left(x,t\right)\psi \left(z\right),$$
(2) outside the beam, for −∞<x<l/2 and l/2<x<∞

(2) $$u_{x}=0,\;u_{y}=0,\;w\left(x,y,z,t\right)=w\left(x,t\right)\psi \left(z\right)\varphi \left(y\right),$$

where $u_{x}$ and $u_{y}$ are the components of horizontal displacement, w is the vertical displacement, $\psi \left(z\right)$ defines the variation of displacements in the vertical direction, and the function $\varphi \left(y\right)$ defines the variation of displacements in the y direction.

The dynamic reaction of the inertial Vlasov foundation to the beam $r\left(x,t\right)$ is given by the equation:

(3) $$r\left(x,t\right)=k_{s}w\left(x,t\right)-c_{s}\frac{\partial^{2}w\left(x,t\right)}{\partial x^{2}}+m_{s}\frac{\partial^{2}w\left(x,t\right)}{\partial t^{2}},$$

where $w\left(x,t\right)$ is the beam deflection; coefficient $k_{s}$ describes the elastic settlement of the foundation and corresponds to the elasticity coefficient of the Winkler model; coefficient $c_{s}$ determines the effect of shear in the soil and is, therefore, a measure of the load transfer to the ground in the vicinity of its application; and $m_{s}$ represents the foundation inertia. The coefficients defining the foundation can be determined using the equations:

(4) $$\begin{array}{c} k_{s}=\frac{E_{0}s_{11}}{1-\nu_{0}^{2}},\;c_{s}=\frac{E_{0}r_{11}}{4\left(1+\nu_{0}\right)},\;m_{s}={\tilde{m}}_{s}b\int_{0}^{H}\psi^{2}\left(z\right)dz,\\ E_{0}=\frac{E_{s}}{1-\nu_{s}^{2}},\;\nu_{0}=\frac{\nu_{s}}{1-\nu_{s}},\;{\tilde{m}}_{s}=\frac{\gamma_{s}}{g},\\ s_{11}=b{\int_{0}^{H}\left[\frac{\partial \psi \left(z\right)}{\partial z}\right]}^{2}dz,\;r_{11}=b\int_{0}^{H}\psi^{2}\left(z\right)dz. \end{array}$$

In Equation (4), b is the width of the beam, ${\tilde{m}}_{s}$ is the mass density of the inertial foundation, $\gamma_{s}$ represents the weight of soil, $E_{s}$ is Young’s modulus of soil, and $\nu_{s}$ is the Poisson’s ratio of the soil layer.

The approximation function $\psi \left(z\right)$ for vertical displacements in the soil layer is assumed to be [22,23,24,25]

(5) $$\psi \left(z\right)=\frac{\mathrm{sh}\gamma \left(1-\frac{z}{H}\right)}{\mathrm{sh}\gamma }.$$

The coefficient γ in Equation (5) can be determined using an iterative procedure or by the method proposed by Ting in their paper [40]. The function $\psi \left(z\right)=1-\frac{z}{H}$ can be assumed for a thin soil layer of thickness H.

## 3. Solution to the Problem

### 3.1. Bernoulli–Euler Beam on Vlasov Foundation Subjected to a Single Moving Force

**Figure 3:**
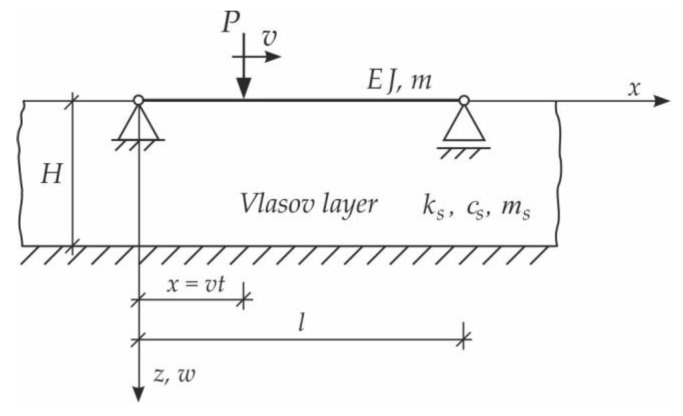
Beam on Vlasov foundation subjected to a moving force.

In the first step, the problem of a single moving force will be solved. We consider vibrations of the simply supported Bernoulli–Euler beam on an inertial Vlasov foundation layer. The beam is subjected to a single force, moving at a constant speed v (Figure 3). The analysis will consider forced vibration when force is on the span, and free vibration when the load is already outside the beam.

After using the soil response function (Equation (3)), the equation of motion of the beam is written in the following form:

(6) $$EJ\frac{\partial^{4}w}{\partial x^{4}}-2c_{s}\frac{\partial^{2}w}{\partial x^{2}}+\left(m+m_{s}\right)\frac{\partial^{2}w}{\partial t^{2}}+k_{s}w=P\delta \left(x-vt\right),$$

where EJ—beam bending stiffness and m—mass per unit length of the beam.

When the Young’s modulus of the beam is variable after function $E\left(z\right)$ or stepwise, in the equation of motion (6), EJ should be replaced with equivalent stiffness $\bar{EJ}$.

(7) $$\bar{EJ}=\int_{-h/2-e}^{h/2-e}b\left(z\right)z^{2}E\left(z\right)dz,$$

where

(8) $$e=\int_{-h/2}^{h/2}E\left(z\right)zdz/\int_{-h/2}^{h/2}E\left(z\right)dz,$$

or for the E stepwise variable

(9) $$e=\sum_{i=1}^{n}\left(E_{i}\int_{-h/2+H_{i-1}}^{-h/2+H_{i}}zdz\right)/\sum_{i=1}^{n}\left(E_{i}\int_{-h/2+H_{i-1}}^{-h/2+H_{i}}dz\right),\;H_{i}=\sum_{j=1}^{i}h_{j}.$$

Vertical displacements w then refer to the beam’s neutral axis, which is set off by e relative to the uniform beam’s neutral axis.

The boundary conditions of the considered beam are as follows:

(10) $${\left.w\left(x,t\right)\right|}_{x=0}=0,\;{\left.w\left(x,t\right)\right|}_{x=l}=0,\;{\left.\frac{\partial^{2}w\left(x,t\right)}{\partial x^{2}}\right|}_{x=0}=0,\;{\left.\frac{\partial^{2}w\left(x,t\right)}{\partial x^{2}}\right|}_{x=l}=0.$$

The solution to Equation (6) is assumed to be in the form of Fourier series, satisfying the boundary conditions of the beam given in Equation (10)

(11) $$w\left(x,t\right)=\sum_{n=1}^{\infty }T_{n}\left(t\right)\sin\frac{n\pi }{l}x.$$

The moving force P is decomposed into Fourier series

(12) $$P\delta \left(x-vt\right)=\sum_{n=1}^{\infty }Q_{n}\left(t\right)\sin\frac{n\pi }{l}x,$$

which yields

(13) $$Q_{n}\left(t\right)=\frac{2P}{l}\sin\frac{n\pi }{l}vt\sin\frac{n\pi }{l}x.$$

Substituting Equations (11) and (13) into Equation (6) means that the equation of the beam motion is

(14) $$\left[{\ddot{T}}_{n}\left(t\right)+\frac{\lambda_{n}^{4}EJ+2c_{s}\lambda_{n}^{2}+k_{s}}{m+m_{s}}T_{n}\left(t\right)-\frac{2P}{\left(m+m_{s}\right)l}\sin\lambda_{n}vt\right]\sin\lambda_{n}x=0,$$

where $\lambda_{n}=\frac{n\pi }{l}$.

Taking into account the eigenfunction $\sin\frac{n\pi }{l}x\neq 0$ yields the time-dependent differential equation of second order

(15) $${\ddot{T}}_{n}\left(t\right)+\omega_{n}^{2}T_{n}\left(t\right)=\frac{2P}{\left(m+m_{s}\right)l}\sin\lambda_{n}vt,$$

where $\omega_{n}^{2}=\frac{\lambda_{n}^{4}EJ+2c_{s}\lambda_{n}^{2}+k_{s}}{m+m_{s}}$, and $\omega_{n}$ is a frequency of the beam on the three-parameter inertial foundation.

Subsequent to zero initial conditions:

(16) $${\left.w\left(x,t\right)\right|}_{t=0}=0,\;\;{\left.\frac{\partial w\left(x,t\right)}{\partial t}\right|}_{t=0}=0,$$

the following function describes the beam deflection:

(17) $$w\left(x,t\right)=\frac{2P}{\left(m+m_{s}\right)l}\sum_{n=1}^{\infty }\frac{1}{\omega_{n}\left(\alpha_{n}^{2}-\omega_{n}^{2}\right)}\sin\lambda_{n}x\left(\alpha_{n}\sin\omega_{n}t-\omega_{n}\sin\alpha_{n}t\right),$$

where $\alpha_{n}=\frac{n\pi }{l}v$.

Equation (17) is valid when the moving force is on the beam, i.e., for $vt\in \langle 0,l\rangle$, and is valid at the load velocity $v<v_{cr}$. The critical velocity $v_{cr}$ can be easily determined by the assumption that the denominator in the trigonometric series (Equation (17)) is equal to zero:

(18) $$\alpha_{n}^{2}=\omega_{n}^{2}\;\Rightarrow \;v_{cr}=\sqrt{\frac{{\left(\frac{n\pi }{l}\right)}^{2}EJ+2c_{s}}{m+m_{s}}+\frac{k_{s}l^{2}}{{\left(n\pi \right)}^{2}\left(m+m_{s}\right)}}.$$

After the force descends from the beam (vt>l), free vibration occurs. The equation of beam motion is then a homogeneous equation with the initial conditions, resulting from the solution in Equation (17) for $t=\frac{l}{v}$

(19) $${\ddot{\bar{T}}}_{n}\left(t\right)+\omega_{n}^{2}{\bar{T}}_{n}\left(t\right)=0,\;\mathrm{for}\;vt>l.$$

The solution to Equation (19) is written as follows

(20) $$\bar{w}\left(x,t\right)=\sum_{n=1}^{\infty }\left(K_{n}\cos\omega_{n}t+L_{n}\sin\omega_{n}t\right).$$

The two constants $K_{n}$ and $L_{n}$ are found using the initial conditions for the time the force leaves the beam ($t=\frac{l}{v}$):

(21) $${\left.w\left(x,t\right)\right|}_{t=\frac{l}{v}}={\left.\bar{w}\left(x,t\right)\right|}_{t=\frac{l}{v}},\;{\left.\frac{\partial w\left(x,t\right)}{\partial t}\right|}_{t=\frac{l}{v}}={\left.\frac{\partial \bar{w}\left(x,t\right)}{\partial t}\right|}_{t=\frac{l}{v}}.$$

**Figure 4:**
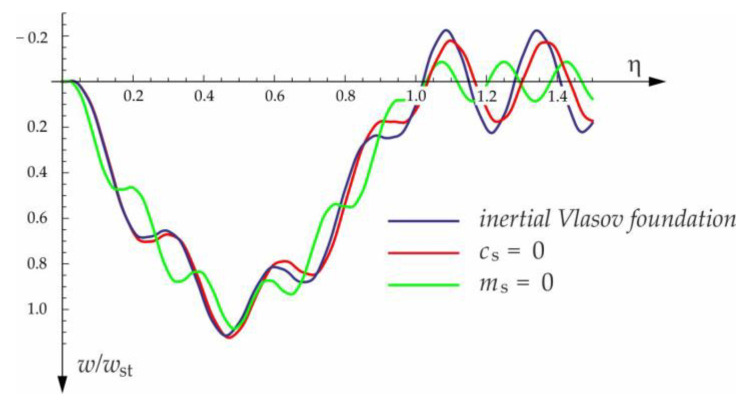
Dynamic deflection of the midpoint of the beam on the foundation subjected to the force moving at v=50 m/s.

**Figure 5:**
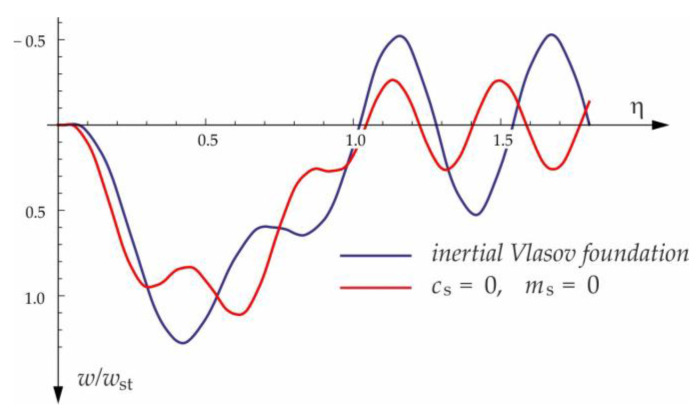
Foundation parameters’ effect on beam midpoint deflection, v=100 m/s.

**Figure 6:**
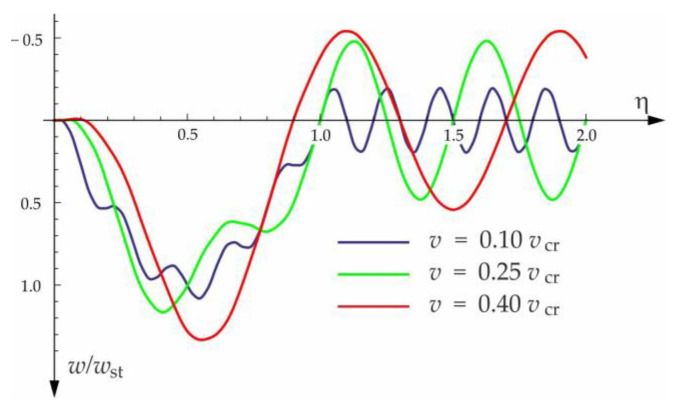
Influence of moving force velocity on the deflection of the midpoint of a beam on three-parameter inertial Vlasov foundation.

**Figure 7:**
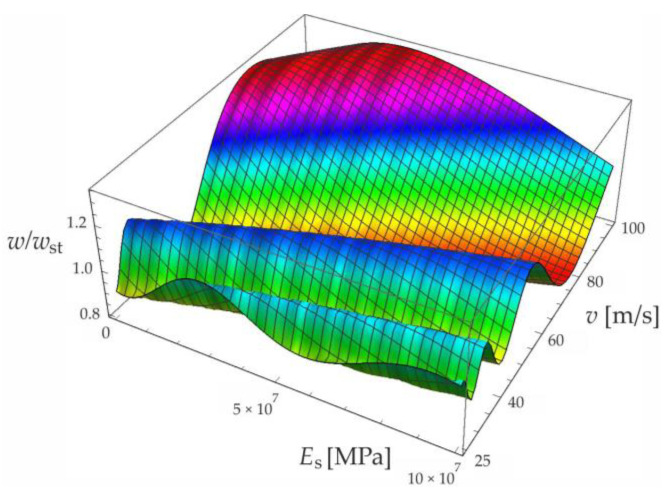
Effect of moving force velocity and a foundation’s Young’s modulus on the deflection of the midpoint of a simply supported beam under forced vibration.

**Figure 8:**
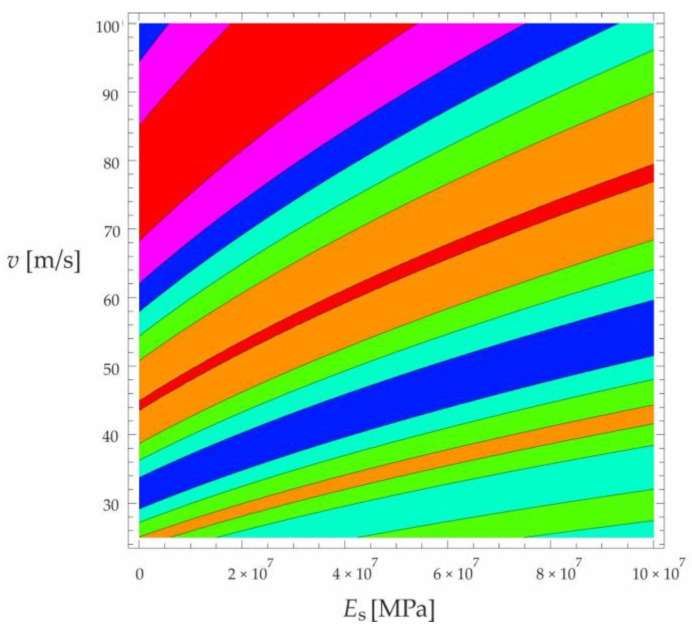
Effect of moving force velocity and a foundation’s Young’s modulus on the deflection of the midpoint of a simply supported beam under forced vibration—contour map corresponding to the diagram in Figure 7.

Based on the above solutions, obtained by the analytical method, the vibrations of a simply supported beam forced by a single force moving along the beam at a constant speed v were determined, as well as the free vibrations of the beam after the load left the structure. The results of the problem are presented in Figure 4, Figure 5, Figure 6, Figure 7 and Figure 8.

The calculations were provided according to the following assumptions: the force magnitude is P=57.5 kN, the beam length is l=5 m, the cross-section is rectangular (with dimensions b×h=0.25×0.47 m), a uniformly distributed mass of the beam is *m* = 282 kg/m, and the Young’s modulus of the beam is assumed to be constant with a value E=34 GPa. 

The beam rests on a layer of inertial foundation where thickness H=1.5 m. The coefficients of the three-parameter Vlasov foundation ($k_{s}$, $c_{s}$ and $m_{s}$) were determined, based on Equation (4). The following mechanical characteristics of the soil were adopted in the calculations:

- Young’s modulus $E_{s}=100\;\mathrm{MPa}$;
- Poisson’s ratio $\nu_{s}=0.30$;
- specific gravity of the soil $\gamma_{s}=26\;\mathrm{kN}/m^{3}$.

Since the soil layer is thin, the function $\psi \left(z\right)$ describing the displacement field is assumed in the form $\psi \left(z\right)=1-\frac{z}{H}$.

The presented solutions and numerical examples concern speeds lower than the critical velocity. A dimensionless coefficient $\eta =\frac{vt}{l}$ was introduced into the calculations. In the diagrams, deflection referred to the static displacement of the beam’s midpoint $w_{st}$.

Figure 4, Figure 5, Figure 6, Figure 7 and Figure 8 show vibrations of the midpoint of the beam, forced by a force moving at uniform speed (0≤η≤1) and free vibrations after the load left the structure (η>1). The impact of the foundation coefficients on the beam deflection is exemplified in Figure 4 and Figure 5. Figure 6 illustrates the influence of a moving force’s velocity on the deflection of the midpoint of the beam on the three-parameter inertial Vlasov foundation. The effect of the load velocity and Young’s modulus on the deflection at the midpoint of a simply supported beam is shown in Figure 7 and Figure 8.

### 3.2. Bernoulli–Euler Beam on Vlasov–Leontiev Foundation Subjected to a Group of Two Moving Forces

**Figure 9:**
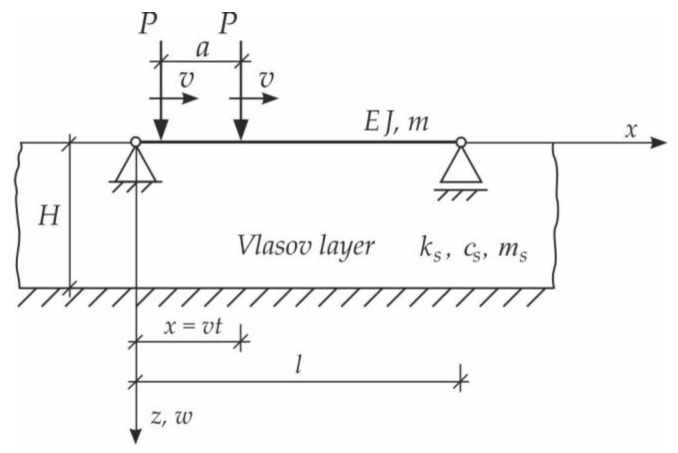
Beam on Vlasov foundation subjected to a group of two moving forces.

We now consider a beam subjected to two forces moving along the span at a constant speed. The forces are separated by a constant distance a from each other (Figure 9). In this case, the load on the beam is described by the formula

(22) $$q\left(x,t\right)=P\delta \left(x-vt\right)+P\delta \left[x-\left(vt-a\right)\right].$$

We will analyse the forced and free vibrations after one and then two forces descend from the beam.

According to the notation in Figure 9, the partial differential equation of the undamped vibration of the beam subjected to the group of two forces is as follows:

(23) $$EJ\frac{\partial^{4}w}{\partial x^{4}}-2c_{s}\frac{\partial^{2}w}{\partial x^{2}}+\left(m+m_{s}\right)\frac{\partial^{2}w}{\partial t^{2}}+k_{s}w=P\delta \left(x-vt\right)+P\delta \left[x-\left(vt-a\right)\right].$$

The solution to the equation of motion (Equation (23)) can be written using Duhamel integrals

(24) $$w\left(x,t\right)=\frac{2P}{\left(m+m_{s}\right)l}\sum_{n=1}^{\infty }\frac{1}{\omega_{n}}\sin\frac{n\pi x}{l}\left\{\int_{0}^{t}\sin\alpha_{n}\tau \sin\left[\omega_{n}\left(t-\tau \right)\right]d\tau +\int_{0}^{t}\sin\left(\alpha_{n}\tau -\frac{n\pi a}{l}\right)\sin\left[\omega_{n}\left(t-\tau \right)\right]d\tau \right\},$$

or in the equivalent form

(25) $$\begin{array}{c} w\left(x,t\right)=\frac{2P}{\left(m+m_{s}\right)l}\sum_{n=1}^{\infty }\frac{1}{\omega_{n}\left(\alpha_{n}^{2}-\omega_{n}^{2}\right)}\sin\frac{n\pi x}{l}\left[\alpha_{n}\sin\omega_{n}t\cos\lambda_{n}a-\omega_{n}\cos\omega_{n}t\sin\lambda_{n}a+\right.\\ \left.-\omega_{n}\sin\left(\alpha_{n}t-\lambda_{n}a\right)+\alpha_{n}\sin\omega_{n}t-\omega_{n}\sin\alpha_{n}t\right]. \end{array}$$

The notation in Equations (23)–(25) is the same as in the case of the beam subjected to a single force. The equation of motion (Equation (23)) and functions (24) and (25) are valid when the load is on the beam. To determine the free vibration, the procedure is the same as in the case of a single force load (see Section 3.1).

**Figure 10:**
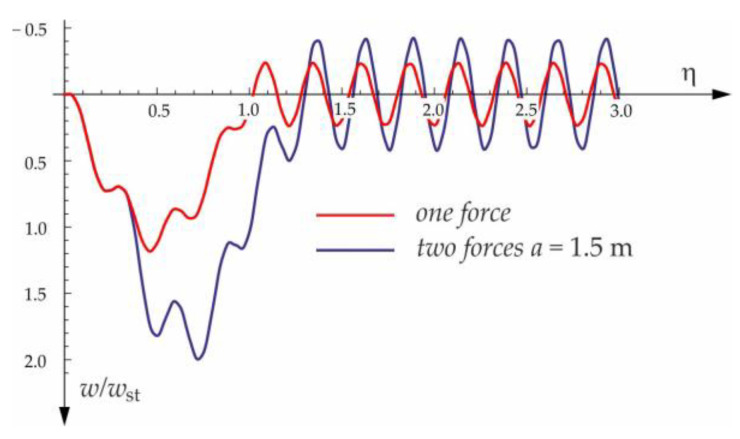
Vibration of midpoint of the beam on three-parameter Vlasov foundation subjected to one force and two forces moving at speed v=50 m/s.

**Figure 11:**
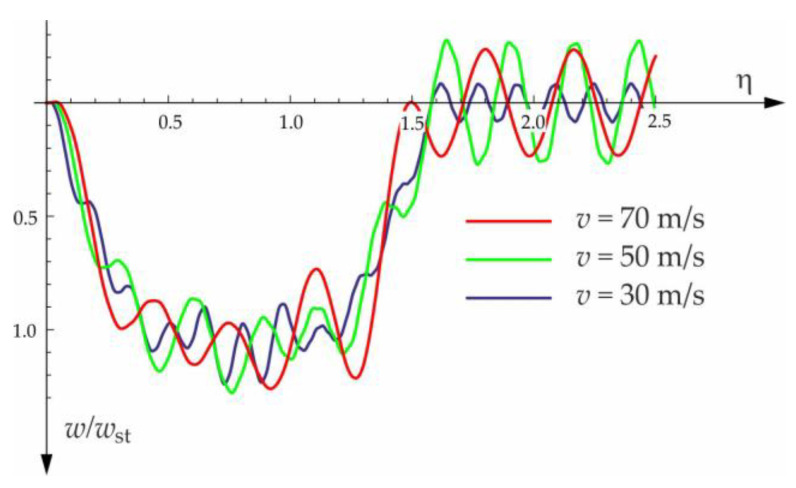
Influence of moving force velocity on deflection of midpoint of the beam on three-parameter Vlasov foundation subjected to a group of two forces moving at a constant distance a=3 m.

**Figure 12:**
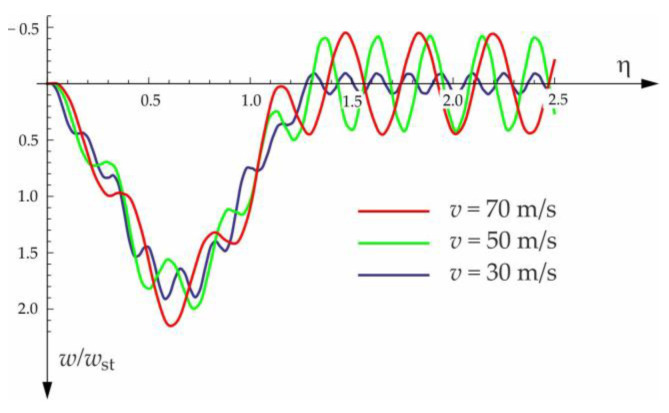
Influence of moving force velocity on the deflection of the midpoint of the beam on three-parameter Vlasov foundation subjected to the group of two forces moving at a constant distance a=1.5 m.

**Figure 13:**
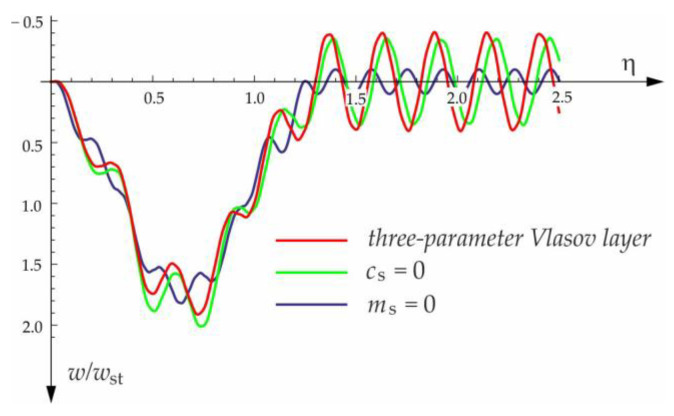
Foundation parameters’ effect on the beam midpoint deflection. Beam subjected to the group of two forces moving at a constant distance of a=1.5 m and a velocity of v=50 m/s.

The calculated results, after programming the formulas for forced and free vibrations, are presented graphically in the diagrams below (Figure 10, Figure 11, Figure 12 and Figure 13). Calculations are provided for the same assumptions, concerning the dimensions and mechanical properties of the beam and the soil layer, as presented in Section 3.1.

### 3.3. Bernoulli–Euler Beam on Vlasov Foundation Subjected to a Group of Three Moving Forces

**Figure 14:**
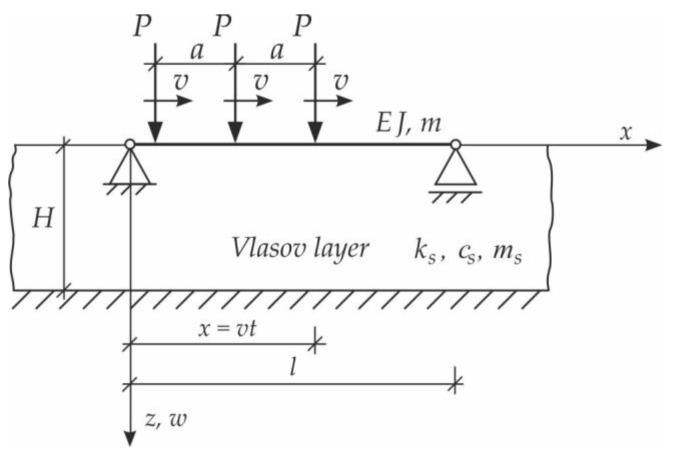
Beam on Vlasov foundation subjected to a group of three moving forces spaced at a constant distance a.

In the case of three moving forces, spaced at a constant distance a and moving at a uniform velocity v, according to Figure 14, the equation of the beam forced vibrations is given by Equation (26):

(26) $$EJ\frac{\partial^{4}w}{\partial x^{4}}-2c_{s}\frac{\partial^{2}w}{\partial x^{2}}+\left(m+m_{s}\right)\frac{\partial^{2}w}{\partial t^{2}}+k_{s}w=P\delta \left(x-vt\right)+P\delta \left[x-\left(vt-a\right)\right]+P\delta \left[x-\left(vt-2a\right)\right].$$

Solving the problem for the group of three forces comes down to solving three Duhamel integrals

(27) $$\begin{array}{c} w\left(x,t\right)=\frac{2P}{\left(m+m_{s}\right)l}\sum_{n=1}^{\infty }\frac{1}{\omega_{n}}\sin\frac{n\pi x}{l}\left\{\int_{0}^{t}\sin\alpha_{n}\tau \sin\left[\omega_{n}\left(t-\tau \right)\right]d\tau +\int_{0}^{t}\sin\left(\alpha_{n}\tau -\frac{n\pi a}{l}\right)\sin\left[\omega_{n}\left(t-\tau \right)\right]d\tau +\right.\\ \left.+\int_{0}^{t}\sin\left(\alpha_{n}\tau -\frac{2n\pi a}{l}\right)\sin\left[\omega_{n}\left(t-\tau \right)\right]d\tau \right\}. \end{array}$$

The function describing the deflection of the beam, after solving Duhamel integrals, can be written as follows:

(28) $$\begin{array}{c} w\left(x,t\right)=\frac{2P}{\left(m+m_{s}\right)l}\sum_{n=1}^{\infty }\frac{1}{\omega_{n}\left(\alpha_{n}^{2}-\omega_{n}^{2}\right)}\sin\frac{n\pi x}{l}\left[v_{n}\sin\omega_{n}t\cos\lambda_{n}a-\omega_{n}\cos\omega_{n}t\sin\lambda_{n}a+\right.\\ -\omega_{n}\sin\left(v_{n}t-\lambda_{n}a\right)+\alpha_{n}\sin\omega_{n}t-\omega_{n}\sin\alpha_{n}t+\alpha_{n}\cos2\lambda_{n}a\sin\omega_{n}t+\\ \left.+\omega_{n}\sin\left(2\lambda_{n}a-\alpha_{n}t\right)-2\sin2\lambda_{n}a\cos\omega_{n}t\right], \end{array}$$

where $\lambda_{n}=\frac{n\pi }{l}$, $\alpha_{n}=\lambda_{n}v$.

The Equations (27) and (28) describe forced vibrations, when the load is on the beam. Free vibrations, after the load has descended from the beam, are determined in the same way as in the two previous cases, when the beam is loaded with a single force or a group of two forces (see Section 3.1). The calculation results for forced and free vibrations of the beam under three forces, considering damping, are shown graphically in Section 4.

## 4. Damped Vibration

When damped vibrations, according to the Kelvin–Voigt model, of the beam on the inertial Vlasov foundation, due to a group of three forces, are considered, the following equation describes the motion of the beam:

(29) $$\begin{array}{c} EJ\frac{\partial^{4}w}{\partial x^{4}}+c_{i}J\frac{\partial^{5}w}{\partial t\partial x^{4}}-2c_{s}\frac{\partial^{2}w}{\partial x^{2}}+\left(m+m_{s}\right)\frac{\partial^{2}w}{\partial t^{2}}+c_{e}\frac{\partial w}{\partial t}+k_{s}w=\\ =P\delta \left(x-vt\right)+P\delta \left[x-\left(vt-a\right)\right]+P\delta \left[x-\left(vt-2a\right)\right]. \end{array}$$

In Equation (29), $c_{e}$ and $c_{i}$ are the damping coefficients of the beam. We assume the proportionality of $c_{e}$ to the mass of the beam m, the proportionality of $c_{i}$ to the Young’s modulus E, and define the equivalent damping number $\xi_{n}$:

(30) $$c_{e}=\alpha_{e}m,c_{i}=\alpha_{i}E,\xi_{n}=\frac{1}{2}\left(\frac{\alpha_{e}}{\omega_{n}}+\alpha_{i}\omega_{n}\right).$$

The considered problem may be solved by the Bubnov–Galerkin method.

Function $w\left(x,t\right)$, describing deflection of the beam, is assumed in the form fulfilling the boundary conditions of the simply supported beam

(31) $$w\left(x,t\right)=\sum_{n=1}^{\infty }T_{n}\left(t\right)\sin\frac{n\pi x}{l}.$$

Equation (31) and the assumptions for the eigenfunction $\sin\frac{n\pi }{l}x\neq 0$ yield the equation

(32) $${\ddot{T}}_{n}\left(t\right)+2\mu {\dot{T}}_{n}\left(t\right)+\omega_{n}^{2}T_{n}\left(t\right)=\frac{2P}{\left(m+m_{s}\right)l}\left[\sin\frac{n\pi vt}{l}+\sin\frac{n\pi \left(vt-a\right)}{l}+\sin\frac{n\pi \left(vt-2a\right)}{l}\right].$$

In Equation (32), coefficient μ is defined by the following equation:

(33) $$2\mu =\frac{c_{i}J}{m+m_{s}}\lambda_{n}^{2}\left(\lambda_{n}^{2}+\frac{2c_{s}}{EJ}\right)+\frac{c_{e}}{m+m_{s}},$$

where $\lambda_{n}=\frac{n\pi }{l}$.

The natural frequency of vibration of the beam on the three-parameter Vlasov layer $\omega_{n}$ is

(34) $$\omega_{n}=\sqrt{\frac{1}{m+m_{s}}\left\{k_{s}+{\left(\frac{n\pi }{l}\right)}^{2}\left[EJ{\left(\frac{n\pi }{l}\right)}^{2}+2c_{s}\right]\right\}}.$$

The zero initial conditions of the problem

(35) $${\left.w\left(x,t\right)\right|}_{t=0}=0,{\left.\frac{\partial w\left(x,t\right)}{\partial t}\right|}_{t=0}=0,$$

yield the following equation for deflection of the beam on the three-parameter inertial foundation

(36) $$\begin{array}{c} w\left(x,t\right)=\frac{2P}{\left(m+m_{s}\right)l}\sum_{n=1}^{\infty }\frac{1}{{\tilde{\omega }}_{n}}\sin\frac{n\pi x}{l}\left\{\int_{0}^{t}e^{-\mu_{n}\left(t-\tau \right)}\sin\alpha_{n}\tau \sin\left[{\tilde{\omega }}_{n}\left(t-\tau \right)\right]d\tau +\right.\\ +\int_{0}^{t}e^{-\mu_{n}\left(t-\tau \right)}\sin\left(\alpha_{n}\tau -\frac{n\pi a}{l}\right)\sin\left[{\tilde{\omega }}_{n}\left(t-\tau \right)\right]d\tau \left.+\int_{0}^{t}e^{-\mu_{n}\left(t-\tau \right)}\sin\left(\alpha_{n}\tau -\frac{2n\pi a}{l}\right)\sin\left[{\tilde{\omega }}_{n}\left(t-\tau \right)\right]d\tau \right\}. \end{array}$$

In Equation (36), ${\tilde{\omega }}_{n}$ is a frequency of the beam-damped vibration

(37) $${\tilde{\omega }}_{n}=\omega_{n}\sqrt{1-\xi_{n}},$$

where $\xi_{n}$ is an equivalent damping number given by Equation (30).

The integrals in Equation (36) can be solved easily. Since the equations are extensive, we do not quote function $w\left(x,t\right)$ after integration.

**Figure 15:**
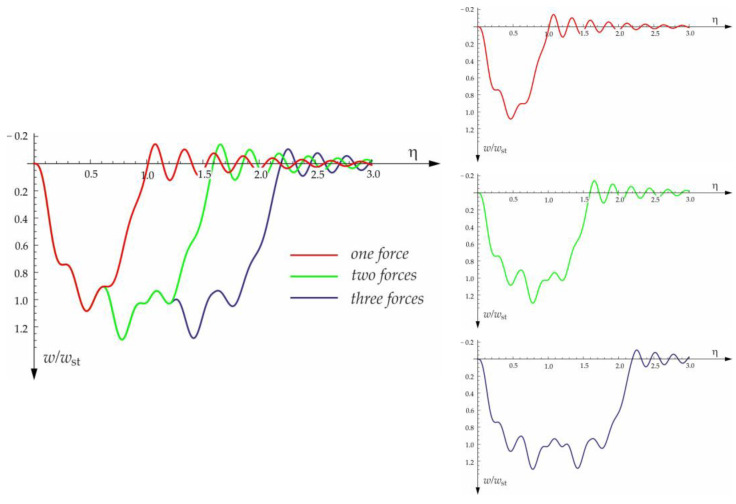
Vibration of midpoint of the beam on three-parameter Vlasov foundation subjected to one force, two forces, and three forces moving at a speed of v=50 m/s, a distance of a=3 m, and a damping number of ξ=0.05.

**Figure 16:**
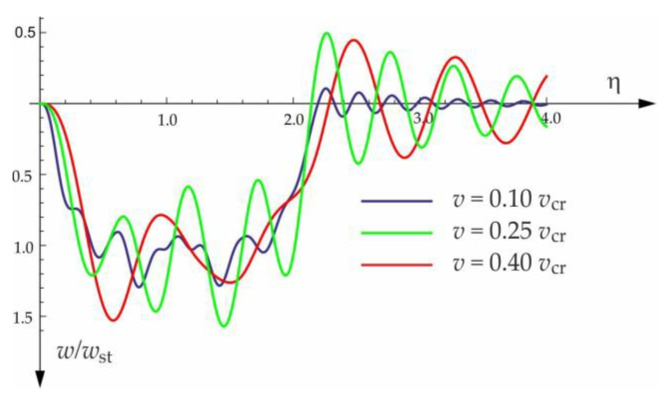
Influence of moving forces’ velocity on deflection of midpoint of the beam on three-parameter Vlasov foundation subjected to the group of three forces moving at a distance of a=3 m and a damping number of ξ=0.05.

**Figure 17:**
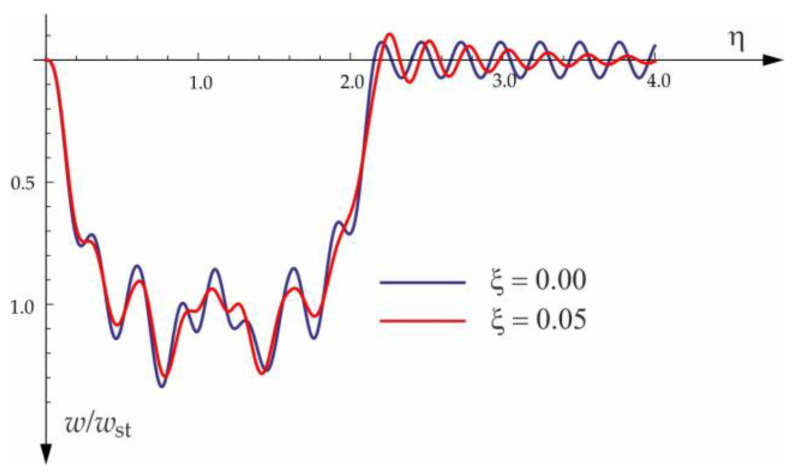
Damping influence on deflection of midpoint of the beam on three-parameter Vlasov foundation subjected to the group of three forces moving at a velocity of v=50 m/s and a distance of a=3 m.

**Figure 18:**
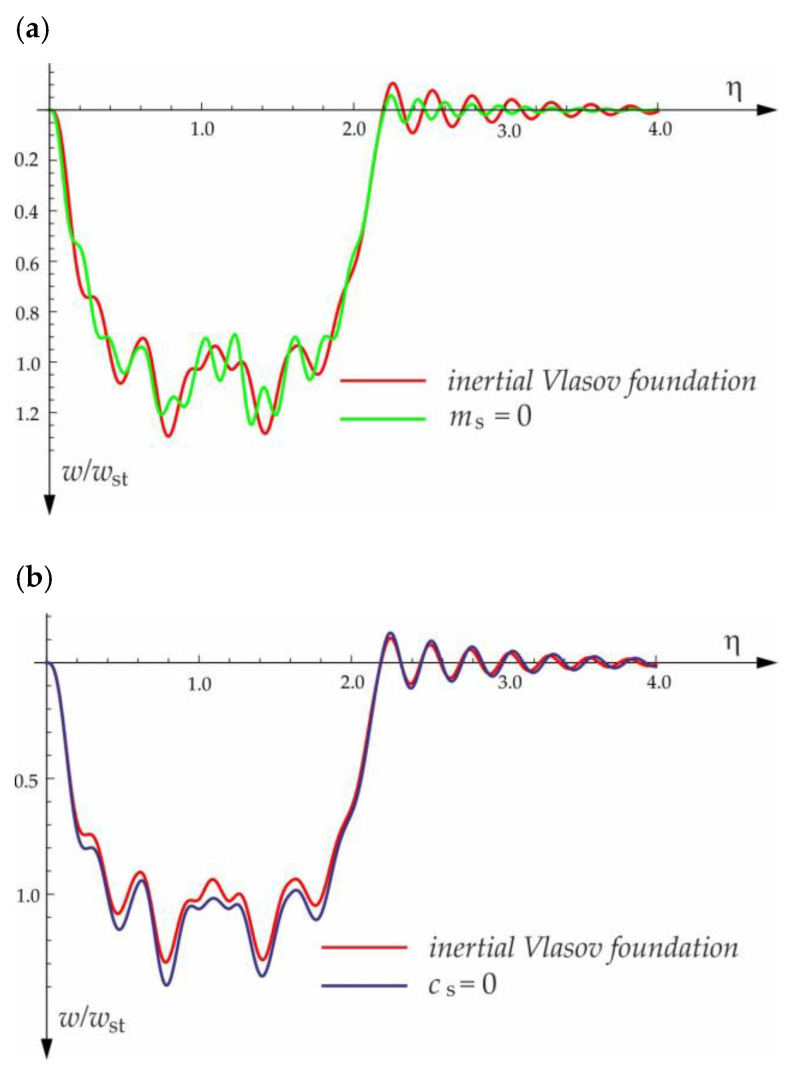
Influence of foundation parameters on the deflection of the midpoint of the beam subjected to the group of three forces moving at a velocity of v=50 m/s and a distance of a=3 m: (**a**) mass coefficient, (**b**) shear coefficient.

The free vibration of the beam can be found in the same way as the beam under a single force (see Section 3.1). Solutions for forced and free vibrations of the beam under a group of three forces are presented graphically in Figure 15, Figure 16, Figure 17 and Figure 18. As before, the dimensionless coefficient $\eta =\frac{vt}{l}$ is introduced into the calculations. In the diagrams, deflection is referred to the static displacement of the beam’s midpoint $w_{st}$ under the action of one force. Calculations are provided for the same assumptions concerning the dimensions and mechanical properties of the beam and the soil layer, as presented in Section 3.1 and Section 3.2.

## 5. Conclusions

The problem of a beam on a three-parameter inertial foundation under a moving load was discussed in the paper and can be used as a benchmark in the calculation of structures under moving loads caused by road or railroad vehicles. In most cases, in analytical solutions, the authors discuss loading with a single force, a material point, or an oscillator. The solutions presented in this paper helps to assess the influence of the group of moving forces and their distance on the displacement of the beam on the Vlasov foundation. Based on the conducted analysis, it can be seen that the appropriate selection of the soil parameters helps to reduce the beam deflections. The given solutions allow us to simply carry out a preliminary selection of the base parameters on which the engineering structure rests. 

The function ψ (5), which decreases deep into the layer and the foundation coefficients, depends on the dimensionless parameter γ. In turn, the parameter γ depends on the thickness of the layer, the Poisson number, and the deflection of the beam and the derivative of this deflection. In order to solve a specific problem in the statics or dynamics of beams on the modified Vlasov foundation, iterative procedures should be used or the foundation parameters should be determined using the method proposed by Ting [40].

The paper analyses the influence of individual foundation coefficients on the beam deflection values caused by one, two, or three moving forces. The coefficient determining the foundation inertia has a significant influence on the dynamic deflection of the beam. Taking shear into account in the Vlasov foundation model has little effect on the dynamic deflections of the beam. The equivalent damping number introduced into the Kelvin–Voigt model takes into account the structure damping and mass damping of the beam.

## Data Availability

Not applicable.
